# Inferior mesenteric vein serves as an alternative guide for transection of the pancreatic body during pancreaticoduodenectomy with concomitant vascular resection: a comparative study evaluating perioperative outcomes

**DOI:** 10.1186/s40001-014-0042-z

**Published:** 2014-08-21

**Authors:** Yonghua Chen, Xing Wang, Nengwen Ke, Gang Mai, Xubao Liu

**Affiliations:** 1 Department of Hepatobiliopancreatic Surgery, West China Hospital, Sichuan University, GuoXue Lane No 37, Chengdu 610041, China

**Keywords:** pancreatic cancer, pancreaticoduodenectomy, Whipple procedure, mesenteric vein resection, inferior mesenteric vein

## Abstract

**Background:**

Tumors of the pancreatic head often involve the superior mesenteric and portal veins. The purpose of this study was to assess perioperative outcomes after pancreaticoduodenectomy (PD) with concomitant vascular resection using the inferior mesenteric vein (IMV) as a guide for transection of the pancreatic body (Whipple at IMV, WATIMV).

**Methods:**

One hundred thirty-seven patients had segmental vein resection during PD between January 2006 and June 2013. Depending on whether the standard approach of creating a tunnel anterior to the mesenterico-portal vein (MPV) axis was achieved for pancreatic transection, patients were subjected to a standard PD with vein resection procedure (s-PD + VR, n = 75) or a modified procedure (m-PD + VR, n = 62). Within the m-PD + VR group, 28 patients underwent the WATIMV procedure, while 34 patients underwent the usual procedure of transection, or ‘central pancreatectomy’ (c-PD + VR).

**Results:**

The volume of intraoperative blood loss and the blood transfusion requirements were significantly greater, and the venous wall invasion and neural invasion frequency were significantly higher in the m-PD + VR group compared with the s-PD + VR group. There were no significant differences in the length of hospitalization, postoperative morbidity, and grades of complications between the two groups. Multivariate logistic regression identified intraoperative blood transfusion (*P* = 0.004) and vascular invasion (*P* = 0.008) as the predictors of postoperative morbidity. Further stratification of the entire cohort of 62 (45%) patients who underwent m-PD + VR showed a higher rate of negative resection margins (96.4%) in the WATIMV group compared with the c-PD + VR group (76.5%) (*P* = 0.06). The volume of intraoperative blood loss (*P* = 0.013), and intraoperative blood transfusion requirements (*P* = 0.07) were significantly greater in the c-PD + VR group compared with the WATIMV group. Furthermore, high intraoperative blood loss and tumor stage were predictive of a positive resection margin.

**Conclusions:**

‘Whipple at the IMV (WATIMV)’ has comparable postoperative morbidity with standard PD + VR. If IMV runs into the splenic vein, it could serve as an alternative guide for transection of the pancreatic body during PD + VR.

## Background

The technique of pancreaticoduodenectomy (PD) has evolved, particularly in relation to the techniques used to achieve negative resection margins. Cancers of the head of the pancreas frequently invade the superior mesenteric and portal veins [1]. Early emphasis was placed on establishing a dissection plane between the anterior surface of the mesenterico-portal vein (MPV) and the posterior surface of the neck of the pancreas. For a long time, diseases involving these veins were considered unresectable. Recently, however, extensive vascular resection has become a standard procedure at major pancreatic surgical centers [2] and has been shown to improve the rates of R0 resection and survival [3] without an increase in postoperative morbidity and mortality [4]. Vein resections usually involve the right lateral portion of the MPV or a cylinder of the superior mesenteric vein (SMV) below its confluence with the splenic vein (SV). Occasionally, the anterior surface of the MPV axis and the posterior surface of the neck of the pancreas are both involved by the tumor or related inflammatory adhesions. This pattern of vein involvement is most likely to occur in tumors of the pancreatic neck or medial aspect of the head of the pancreas. The standard approach of developing a tunnel behind the neck of the pancreas, anterior to the MPV axis, cannot be achieved when these veins are infiltrated with tumor or related inflammatory adhesions, and these tumors are frequently considered unresectable. Previously, we have described an alternative procedure of PD for dealing with these difficult tumors, which we refer to as ‘Whipple at the inferior mesenteric vein (WATIMV)’ [5]. The aim of this study is to evaluate the pattern and to compare it to the pattern in cases with the conventional approach.

## Methods

### Patient characteristics and inclusion criteria

Medical records of patients who underwent PD at West China Hospital between January 2006 and June 2013 were retrospectively reviewed. During this interval, all PD were prospectively entered into a maintained database. A total of 1,053 patients underwent a curative-intent PD for cancer diseases. Of these, 178 (16.9%) with locally advanced disease had SMV-PV resection and reconstruction as part of their procedure. Thirty-six patients (20.9%) had partial lateral venorrhaphy and five patients (2.8%) had a concomitant arterial resection. These cases were excluded, leaving a study population of 137 (77%) who had segmental vein resection, including portal vein (PV) resection in 15 patients, SMV resection in 85 patients, and MPV-SV confluence resection in 37 patients. Depending on whether or not the standard approach of creating a tunnel anterior to the MPV axis was achieved, patients were subjected to the standard procedure with concomitant vascular resection (s-PD + VR, n = 75) or a modified procedure (m-PD + VR, n = 62), according to the surgeons’ clinical and operative notes. Within the m-PD + VR group, 28 patients underwent a WATIMV procedure, while 34 patients underwent the usual procedure of transection, or ‘central pancreatectomy’ (c-PD + VR). The patients’ characteristics, blood loss, blood transfusion, postoperative mortality and morbidity, and histological results were collected from the medical records. Perioperative mortality was defined as in-hospital mortality and death within 60 days after discharge of patient. This study followed the ethical guidelines of the Helsinki Declaration of 1975 (revised in 1983).

### Surgical technique

**Figure 1:**
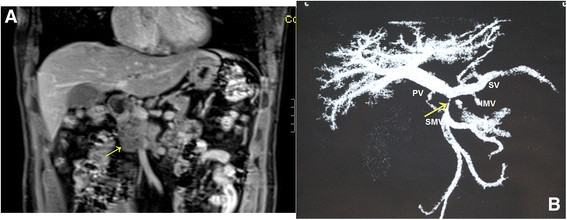
**Magnetic resonance imaging** (**MRI) of a patient with segmental vein occlusion. A)** A mass with complete occlusion of the superior mesenteric vein (SMV) (arrow); **B)** A multiple planar volume reconstruction image showed complete occlusion of the SMV with a venous collateral (arrow); the inferior mesenteric vein (IMV) inserted into the splenic vein (SV). PV, portal vein.

**Figure 2:**
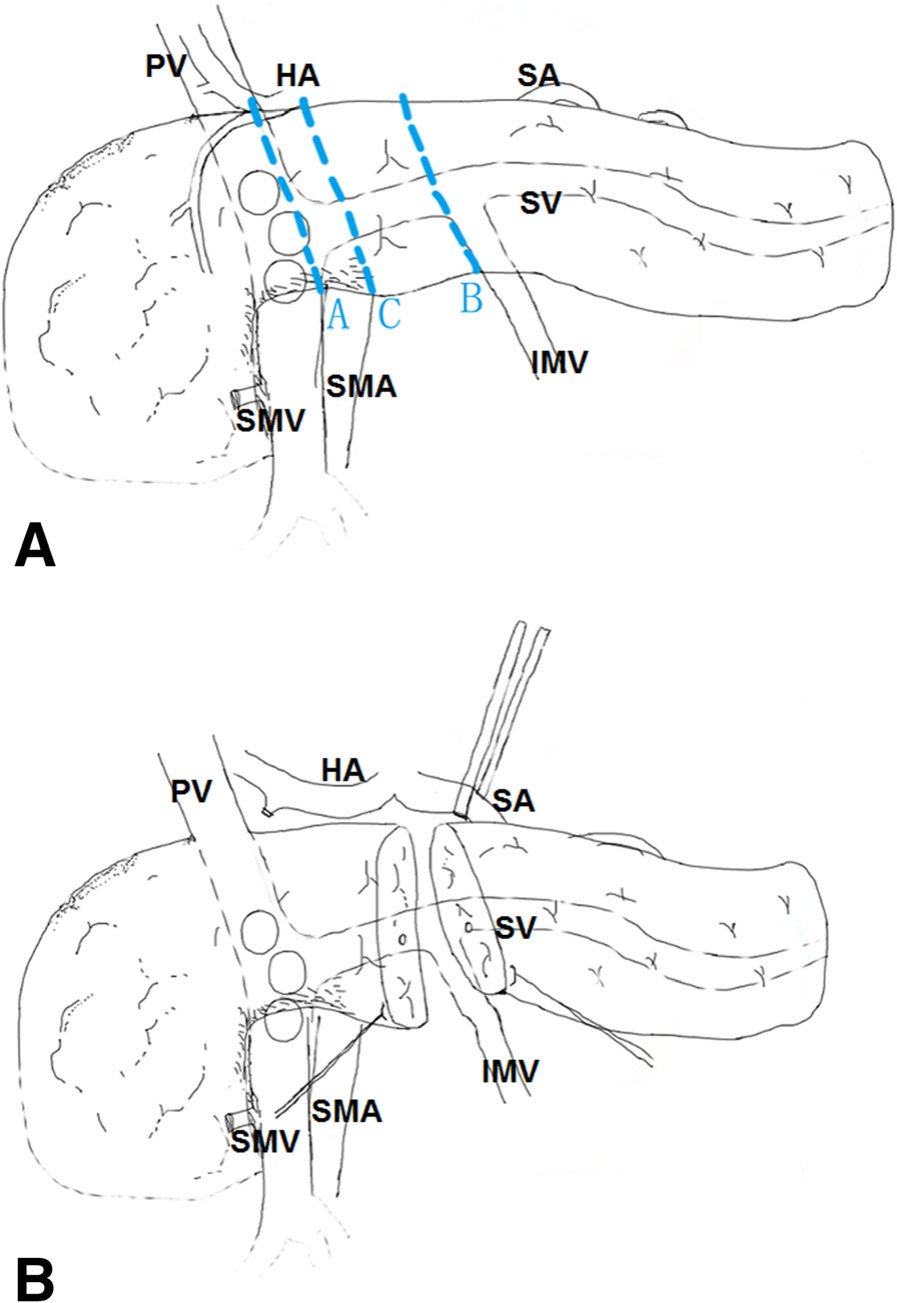
**Schematic diagram. A)** Pancreatic tumors involving the anterior of the mesenterico-portal vein **(**MPV) axis (circles); line A indicates the usual plane of transection of the neck of the pancreas in a standard Whipple procedure; line B indicates the plane of transection in an interior mesenteric vein (IMV) procedure; line C indicates the plane of transaction as in the ‘central pancreatectomy’ approach. **B)** The pancreas is transected just to the right of the plane through which the IMV enters the inferior border of the pancreas. (PV, portal vein; HA, hepatic artery; SA, splenic artery; SMV, superior mesenteric vein; SMA, superior mesenteric artery; SV, splenic vein).

Analysis of anatomic variants of mesenteric veins by three-dimensional (3-D) portography was performed using multidetector-row computed tomography (CT) or magnetic resonance imaging (MRI). To be considered for operation, the patients were required to fulfill the following objective CT criteria for tumor respectability [4],[6],[7]: (1) no distant metastases and (2) vein involvement of the MPV without encasement of the nearby arteries. We considered vein involvement below the confluence of its ileal and jejunal tributaries, resulting in the lack of any available vein for anastomosis, a contraindication to resection. This problem was typically defined by preoperative imaging. When tumors involved the MPV (Figure 1), we typically dissected the SMV and created a plane to the left of the PV on to the SV as in the c-PD + VR (Figure 2). Since 2010, the WATIMV approach had been applicable when a tumor involves a cylinder of the MPV (Figure 1) or when the anterior surfaces of the MPV axis and posterior surface of the neck of the pancreas were both involved by the tumor or related inflammatory adhesions.

All patients underwent a standard extended lymph node dissection, which included the hepatic hilum, common hepatic artery, celiac trunk, superior mesenteric artery (SMA), and para-aortic area above the left renal vein. Intraoperative pathological analysis of the pancreatic margin was obtained in all cases. The standard procedure of PD was quite similar in most of the surgical centers and was described elsewhere [2],[8],[9]. Briefly, the modified Kocher maneuver was performed to determine the tumor involvement of the SMA or MPV invasion. If the tumor was not located in the part of the pancreas overlying the superior mesenteric vein, the neck of the pancreas could be divided, as in routine cases (s-PD + VR). If the standard approach of creating a tunnel anterior to the MPV axis could not be achieved, we typically dissected the SMV and created a plane to the left of the PV on to the SV for pancreatic transaction, as in the c-PD + VR (Figure 2A).

**Figure 3:**
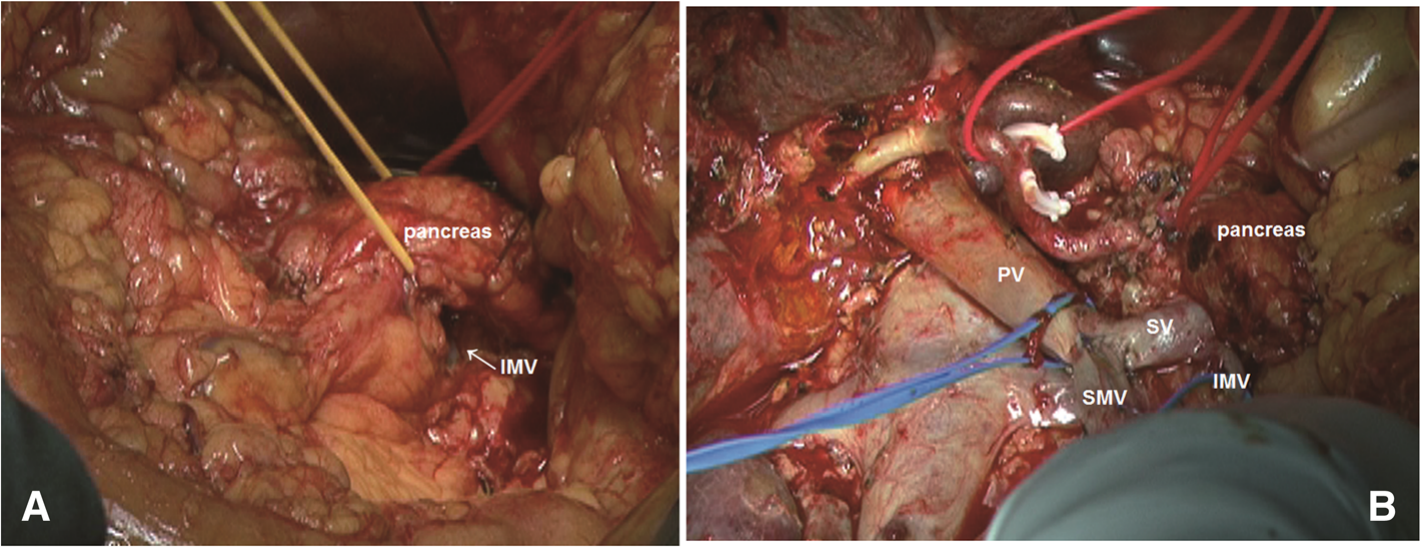
**Intraoperative photographs. A)** creating the tunnel between the anterior surface of the splenic vein (SV) and the posterior surface of the pancreas; the body of the pancreas was encircled with a vessel loop; **B)** The superior mesenteric vein (SMV) has been anastomosed to the portal vein (PV) without a graft. (Reprinted from: Chen YH *et al.*, *J Am Coll Surg* 2013, 217(4):E21-E28., with permission).

The WATIMV approach was used in the present series of patients in a manner similar to that previously described [5]. The key to this technique of MPV resection was the IMV; if drained into the SV, this structure guided the surgeon throughout the procedure (Figure 2). Technical highlights included the following: (1) In this procedure, surgeons were not forced to develop a plane of dissection between the anterior surface of the MPV axis and the posterior surface of the neck of the pancreas when they suspected adherence of the pancreas to the vessels due to tumor invasion or inflammatory reaction; (2) Isolation of the IMV was achieved by tracing up to the IMV-SV confluence for rapid identification, by mobilization of the retroperitoneal attachments down to the level of the body of the pancreas, and by dissection of the tunnel behind the body of pancreas by blunt dissection (Figure 3); and (3) Preservation a part of the MPV-SV confluence when technically possible facilitated the vein anastomosis. Following pancreatic transection, the venous tributaries of SV on the ‘specimen side’ and some of the easily accessible MPV tributaries were ligated and divided. Then, the dissection plane could be continued in a cephalad direction along the anterior aspect of the SMA toward its origin. At this stage, the pancreatic head was held in place only by tumor adhering to the MPV structures. The involved segment of vein could then be clamped proximally and distally at least 0.5 to 1 cm away from the tumor and resected *en bloc* with the specimen. Patients requiring segmental resection of the MPV underwent reconstruction with a primary end-to-end anastomosis whenever possible using 5-0 synthetic, monofilament, nonabsorbable polypropylene suture (Ethicon Inc.) in a running fashion and tied loosely (Figure 3B).

The remainder of the pancreaticoduodenectomy followed our standard protocol of reconstruction [5],[10]. Before closure of the abdomen, a visual inspection was performed to exclude narrowing of the MPV system. The diameter of the anastomosis must be within 75% of the inflow vessel diameter and normal flow in the portal venous system was confirmed by a handheld Doppler ultrasound. Pathologic analysis followed a standardized system for the pathologic evaluation of PD specimens established previously [11]. Tumor stage was determined according to the American Joint Committee on Cancer (AJCC) Cancer Staging Manual, 7th edition [12]. Postoperative complications were recorded and graded according to Dindo and associates [13]. All patients were followed up through our outpatient tumor clinic with physical examination, an abdominal CT scan, Doppler study, and serum blood tests at 3-month intervals for at least half a year.

### Statistical analysis

Continuous data were expressed as means and standard deviations. Proportions were presented as numbers and percentages. Differences in continuous variables were assessed using the Student’s *t*-test. Pearson’s chi-square test was used to compare proportions. Univariate analyses were performed using logistic regression. Factors with *P* <0.10 were included in the multivariate analysis. The final multivariate model was determined using logistic regression with backward selection in order to identify independent predictors of postoperative morbidity and a positive resection margin. Values of *P* less than 0.05 were considered statistically significant. Statistical analyses were performed using SPSS for Windows version 13.0 statistical software.

## Results

Seventy-five patients had a standard PD + VR procedure, and 62 patients underwent modified procedure (m-PD + VR). As shown in Table 1, there was no difference in the preoperative patient characteristics between the s-PD + VR and m-PD + VR groups. The final pathological examination of the resected specimens in both groups showed no differences regarding tumor differentiation, tumor stage, lymph node metastasis and number and site of positive surgical margins. Patients in the m-PD + VR group had an increased intraoperative blood loss (*P* = 0.018), a higher perioperative blood transfusion requirement (*P* = 0.008), and a higher venous wall invasion and neural invasion (both *P* <0.001) compared with those in the s-PD + VR group. The mean length of hospitalization and rates and grades of postoperative complications did not differ between the two groups.

**Table 1:** Clinical characteristics, operative data, postoperative outcomes, and histopathologic features

**Variable**	**Modified procedures**			**Standard procedures (n = 75)**	***P***^**b**^	***P***^**c**^	***P***^**d**^
	**Total (n = 62)**	**WATIMV (n = 28)**	**c-PD (n = 34)**				
Age (yr) (mean (SD))	57.8 ± 10.9	57.2 ± 10.9	58.1 ± 11.1	56.3 ± 10.8	0.398	0.63	0.87
Sex (Male/Female)	38/24	19/9	19/15	51/24	0.41	0.48	0.99
ASA score, n					0.75	0.89	0.96
1	28	13	15	35			
2	24	10	14	25			
3	10	5	5	15			
Operative data							
Length of vein resection, mm (95% CI)	24.19 (22.93 to 25.46)	24.64 (22.67 to 26.61)	23.82(22.1 to 25.54)	20.91 (19.39 to 22.42)	0.002	0.59	0.005
Blood loss, mL (95% CI)	679.8 (557.1 to 802.5)	533.9 (426.7 to 641.2)	800(597.8 to 1002.2)	509.3 (442.7 to 575.9)	0.018	0.013	0.79
Blood loss 500 mL, n	36	13	23	27	0.01	0.012	0.99
Blood transfusion, n	31	10	20	21	0.008	0.07	0.45
Histopathology							
Tumor differentiation, n					0.41	0.97	0.66
Well	0	0	0	2			
Moderate	22	10	12	28			
Poor	40	18	22	45			
Overall stage ^a^, n					0.14	0.58	0.52
1B	0	0	0	3			
2A	34	16	17	47			
2B	29	12	17	24			
3	0	0	0	0			
4	0	0	0	1			
Neural invasion, n	39	18	21	24	0.001	0.84	0.003
Vascular invasion, n	46	21	25	27	0.001	0.895	0.001
Depth of venous wall invasion, No					0.001	0.56	0.001
Adventitia	9	3	6	11			
Tunica media	24	10	14	10			
Intima	13	8	5	6			
Lymph node metastasis, n	29	12	17	25	0.11	0.58	0.37
R1 Margin, n	9	1	8	5	0.13	0.06	0.9
Site of positive margin (no. (%))							
Pancreas	3	0	3	1			
Retroperitoneum	6	1	5	4			
Postoperative data							
Medical complications, n	30	13	17	35	0.84	0.78	0.98
Grade of complications					0.09	0.83	0.057
0	32	15	17	40			
1	9	4	5	13			
2	13	5	8	16			
3	7	4	3	1			
4	0	0	0	4			
5 Mortality, n	1	0	1	1			
Postoperative hospital stay (days) (mean (SD))	17 ± 9.7	15 ± 7.1	18.6 ± 11.3	14.2 ± 5.8	0.55	0.095	0.67

^a^American Joint Committee on Cancer (AJCC) Cancer Staging Manual, 7th edition.

P^b^Patients who underwent standard PD with vein resection procedure (s-PD + VR, n = 75) versus modified procedure (m-PD + VR, n = 62).

P^c^Further stratification of the entire cohort of m-PD + VR group, ‘Whipple at IMV’ procedure (WATIMV n = 28) versus transection procedure as in the ‘central pancreatectomy’ (c-PD + VR, n = 34); P ^d^Patients who underwent standard PD with vein resection procedure (s-PD + VR, n = 75) versus ‘Whipple at IMV’ procedure (WATIMV, n = 28). ASA, American Society of Anesthesiologists; WATIMV, Whipple at inferior mesenteric vein.

A number of variables were analyzed to identify independent predictors of postoperative complications (Table 2). High American Society of Anesthesiologists (ASA) scores, intraoperative blood loss, intraoperative blood transfusion, vascular invasion and tumor differentiation were predictive of postoperative complications on univariate analysis. Multivariate analysis identified ASA scores (*P* = 0.003), intraoperative blood transfusion (*P* = 0.004) and vascular invasion (*P* = 0.008) as the independent variable influencing postoperative complications. There were two deaths (1.46%) within 60 days of operation in this series: acute renal failure (n = 1) in the s-PD + VR group and intraperitoneal hemorrhage (n = 1) in the c-PD + VR group.

**Table 2:** Univariate and multivariate logistic regression analyses of predictors of postoperative complications

**Variables**	**Univariate**^**a**^		**Multivariate**^**a**^	
	**Odds ratio**	***P*** **value**	**Odds ratio**	***P*** **value**
Age, y	0.99 (0.96, 1.03)	0.72		
Sex				
Male	0.61 (0.30, 1.23)	0.17		
Female	1.0			
Resection		0.22		
S-PD	1.0			
c-PD	1.29 (0.57, 2.90)	0.54		
IMV	0.99 (0.42, 2.37)	0.98		
ASA score		0.001		0.003
1	1.0			
2	3.99 (1.81, 8.80)	0.001	3.88 (1.66, 9.10)	0.002
3	4.12 (1.55, 10.95)	0.005	3.68 (1.26. 10.74)	0.017
Blood loss, mL	1.001 (1.0, 1.003)	0.006		NS
Blood loss >500 mL				NS
Yes	2.497 (1.25, 4.98)	0.009		
No	1.0			
Blood transfusion			3.179 (1.43, 7.06)	0.004
Yes	3.88 (1.86, 8.11)	<0.001		
No	1.0			
Neural invasion				NS
Present	1.54 (0.78, 3.03)	0.21		
Absent	1.0			
Vascular invasion			2.87 (1.32, 6.21)	0.008
Present	2.53 (127, 5.05)	0.008		
Absent	1.0			
Lymph node metastasis				NS
Present	1.85 (0.92, 3.698)	0.08		
Absent	1.0			
Tumor differentiation	2.49 (1.25, 4.96)	0.009		NS
Overall stage ^b^	1.69 (0.91, 3.13)	0.096		NS

Values in parentheses are 95% confidence intervals.

^a^Logistic regression.

^b^American Joint Committee on Cancer (AJCC) Cancer Staging Manual, 7th edition. ASA, American Society of Anesthesiologists.

### Subgroup analysis of patients who underwent the Whipple at inferior mesenteric vein procedure

Further stratification of the entire cohort of 62 (45%) patients with aborting to creating a tunnel anterior to the MPV axis as the standard approach showed (Table 1) that demographic characteristics and ASA scores were similar between the two groups. The volume of intraoperative blood loss (*P* = 0.013) and intraoperative blood transfusion requirements (p = 0.07) were significantly greater in the c-PD + VR group compared with the WATIMV group. There were no statistical differences in the length of hospital stay, postoperative morbidity, or grades of complications.

A comparison of tumor characteristics showed that the higher rate of negative resection margins in patients who underwent the WATIMV procedure (96.4%) as compared with those who underwent a c-PD + VR (76.5%) did not reach statistical significance (P = 0.06). In the entire subset of WATIMV patients, only one was identified for whom microscopic involvement (R1) concerned the resection margin of the retroperitoneum. In contrast, eight patients in the c-PD + VR group had microscopic positive margins (R1 resection) that included the retroperitoneal margin (n = 5) or the pancreatic stump (n = 3). Only minimal differences regarding nodal status, perineural invasion, vascular invasion and tumor grading between patients who underwent a WATIMV procedure and a c-PD + VR were assessed (Table 1). Univariate analysis identified c-PD + VR, intraoperative blood loss, intraoperative blood transfusion, vascular invasion, lymph node metastasis and tumor stage as predictors of a likely positive margin; multivariate analysis identified intraoperative blood loss (*P* <0.001) and tumor stage (*P* = 0.017) as the significant predictors of positive resection margin (Table 3).

**Table 3:** Univariate and multivariate logistic regression analyses of predictors of positive resection margin

**Variables**	**Univariate**^**a**^		**Multivariate**^**a**^	
	**Odds ratio**	***P*** **value**	**Odds ratio**	***P*** **value**
Age, y	0.98 (0.94, 1.03)	0.489		
Sex				
Male	0.69 (0.23, 2.12)	0.519		
Female	1.0			
Resection		0.22		NS
S-PD	1.0			
c-PD	4.31 (1.29, 14.37)	0.017		
IMV	0.52 (0.06, 4.65)	0.557		
ASA score		0.721		
1	1.0			
2	1.62 (0.46, 5.56)	0.450		
3	1.58 (0.35, 7.18)	0.553		
Length of vein resection,	1.01 (0.92, 1.11)	0.764		
Blood loss, mL	1.003 (1.001, 1.004)	<0.001	1.003 (1.001, 1.004)	<0.001
Blood loss 500 mL				NS
Yes	3.30 (0.98, 11.11)	0.054		
No	1.0			
Blood transfusion				NS
Yes	5.0 (1.48, 16.91)	0.010		
No	1.0			
Neural invasion				NS
Present	3.30 (0.98, 11.11)	0.054		
Absent	1.0			
Vascular invasion				NS
Present	6.51 (1.40, 30.30)	0.017		
Absent	1.0			
Lymph node metastasis				NS
Present	4.49 (1.33, 15.15)	0.016		
Absent	1.0			
Tumor differentiation	2.43 (0.66, 8.88)	0.181		
Overall stage ^b^	4.20 (1.45, 12.10)	0.008	4.46 (1.31, 15.17)	0.017

Values in parentheses are 95% confidence intervals.

^a^Logistic regression. ^b^American Joint Committee on Cancer (AJCC) Cancer Staging Manual, 7th edition. ASA, American Society of Anesthesiologists.

## Discussion

Infiltration of the MPV is encountered in many patients, especially those with pancreatic head tumors [1]. The management of these tumors represents the most challenging technical aspect of PD. To adhere to the principles of oncologic surgery, this resection should be performed without violating the integrity of the tumor. *En bloc* MPV resection is nowadays considered a safe procedure with mortality and morbidity rates quite similar to the standard PD [14]. The standard approach of creating a tunnel behind the neck of the pancreas, anterior to the MPV axis, cannot be achieved when these veins are infiltrated with tumor or related inflammatory adhesions (Figure 2). If such a procedure were undertaken, the surgery would result in positive margins or the potential for venous injury. Moreover, direct dissection of the tumor from the MPV without using the ‘no touch’ technique could transform a potentially curative resection into a palliative one (by increased risk for intraportal tumor dissemination). Thus, these tumors are frequently considered unresectable. This pattern of vein invasion is most likely to occur in tumors of the neck or medial aspect of the head of the pancreas.

Depending on the site of tumor invasion of the MPV and thus the extent of vein resection required, different technical options for resection and reconstruction are available. When the lesion is adherent to a small part of the lateral or posterior wall of the PV or SMV, dissection of the SMV and the creation of a plane to the left of the PV on to the SV as standard Whipple procedure is possible. On the other hand, when the tumor involves a cylinder of the MPV or when the anterior surfaces of the MPV axis and posterior surface of the neck of the pancreas are both involved by tumor or related inflammatory adhesions, the pancreas must be divided further to the left, abandoning the usual plane of the neck of the pancreas. Many have adopted a selective approach for pancreatic transection, with some authors claiming that the body of the pancreas should be divided with preservation and control of the SV as in the ‘central pancreatectomy’ procedure [12], whereas others describe it as safe to directly cut the pancreas at the body of pancreas and the SV to the left of the PV without isolation and revascularization of the SV [14]. However, routine procedures dividing the body of the pancreas and isolating the SV include complex and troublesome maneuvers. There are many tributaries behind the pancreas that enter the anterior aspect of the SV, and these can be easily damaged during dissection, which is especially difficult in patients with peripancreatic inflammation and adhesions around the body of the pancreas [5],[15]. Moreover, directly cutting the pancreas at the body plane without isolating the SV would result in the potential for venous injury and uncontrollable bleeding and could lead to the necessity for total pancreatectomy and splenectomy. Actually, we had two patients who underwent total pancreatectomy because of SV injury when we used these procedures (data not shown). Strasberg and his colleagues [16] recently described a procedure termed ‘WATSA’ in which the pancreas and SV are divided just to the right of the contact between the splenic artery and the superior border of the pancreas without SV revascularization. However, their routine SV ligation results in the potential development of left-sided portal hypertension and hypertensive gastropathy and/or gastric variceal hemorrhage [17],[18]. When a segment of the PV must be sacrificed, primary end-to-end anastomosis should be made with preservation of all venous branches, including the SV, whenever feasible [14].

Several previous reports have analyzed the paths of the IMV on MRI or multidetector CT. The three most common variants (SV, SMV, and SMV-SV confluence) of the drainage sites of the IMV differ among these reports. The IMV drains into the SV in 42.3% to 56% of Caucasian patients [19],[20] and in 45% to 68.5% of East Asian patients [21],[22]. This is probably due to the fact that the IMV cannot be used as a guiding structure for dissection of the pancreatic body if it runs into the SMV (type SMV). In type SV, the IMV runs along the left side of the SMA and apart from the root of the SMA in most patients [22] and enters the SV at a mean distance (entering point in relation to the portal confluence) of 1.66 cm (range: 0.27 to 3.48) [19], which is chosen as the site of transection of the pancreas to attain negative margin as well as identify, control, and protect the SV. Moreover, there are no direct anterior branches to the IMV-SV confluence, which is the ideal alternative plane to create a tunnel between the anterior surface of veins and the posterior surface of the pancreas by blunt dissection.

An interesting and significant difference in the present study was related to intraoperative blood loss, blood transfusion and positive resection margin. Thus, in the WATIMV group, both intraoperative blood loss and positive resection margin were lower than c-PD + VR group and comparable with standard PD + VR group. This is not surprising because the addition of a major vein resection adds to the complexity of the procedure, during which considerable time is required to dissect and control all portal venous tributaries before resection. Frequently, these tributaries are obstructed due to tumor involvement, and dissection of these fragile collateral veins may lead to significant blood loss. Reduced blood loss and positive resection margin in the WATIMV group could be attributed to the following: (1) In these surgeries the development of the usual plane of transection as standard procedure was never forced when adherence of the pancreas to the vessels due to tumor invasion or inflammatory reaction was suspected, because when this maneuver is unsuccessful, the surgeon may left with either a grossly positive margin or an inadvertent venotomy; (2) In order to attain a negative margin and identify and protect the SV, the pancreas was divided further to the left as the point where the right of the IMV enters into the inferior border of the pancreas with a mean distance of 1.66 cm [19], which meant abandoning the usual plane of the neck of the pancreas; and (3) In order to avoiding venous injury and uncontrollable bleeding, identification of the IMV and whether it drained into the SV served as a guide to securely create the tunnel between the anterior surface of SV and the posterior surface of pancreas by blunt dissection as a standard procedure, because there are no direct anterior branches to the IMV-SV confluence. Venous injury often results in uncontrolled hemorrhage and the necessity for rapid removal of the tumor without proper attention to the SMA dissection. Therefore, it is easy to appreciate how such cases may result in a positive margin. WATIMV is an alternative approach to the specific problem of the involvement of the anterior surface of the MPV axis by tumor. Furthermore, this technique may be very helpful when there is severe inflammation around the veins and when it is difficult to distinguish the scar from the cancer intraoperatively [5]. In a standard PD procedure, venous injury, uncontrollable bleeding from within the tunnel behind the neck of the pancreas, and transection across the tumor are not rare events. This new approach can provide controlled rescue.

## Conclusions

Our data suggest that major pancreatic surgery can be safely combined with *en bloc* VR in case of suspected or evidenced vascular invasion. If IMV runs into the SV, it could serve as an alternative guide for transection of the pancreatic body during PD + VR to avoid the potential for venous injury and uncontrollable bleeding. Although the short-term benefits of offering patients a resection have been shown, it will be important to assess the long-term outcomes in order to understand the true impact of these complex vein resections in this group of patients.

## Abbreviations

ASA: American Society of Anesthesiologists

c-PD + VR: central pancreatectomy

CT: computed tomography

IMV: inferior mesenteric vein

m-PD: modified pancreaticoduodenectomy

MPV: mesenterico-portal vein

MRI: magnetic resonance imaging

PD: pancreaticoduodenectomy

PV: portal vein

SMV: superior mesenteric vein

s-PD + VR: standard pancreaticoduodenectomy with vein resection

SV: splenic vein

VR: vein resection

WATIMV: Whipple at inferior mesenteric vein

## Competing interests

The authors have no conflict of interests to declare. No benefits in any form have been received or will be received from a commercial party related directly or indirectly to the subject of this article.

## Authors’ contributions

LXB, CYH and MG designed the research; LXB MG, CYH, WX and KNW performed the research; CYH, WX, and KNW analyzed the data; and CYH, LXB and MG wrote the paper. All authors read and approved the final manuscript.
